# Differences in morphology and visual function of myelin oligodendrocyte glycoprotein antibody and multiple sclerosis associated optic neuritis

**DOI:** 10.1007/s00415-020-10097-x

**Published:** 2020-08-12

**Authors:** Rino Vicini, Dominik Brügger, Mathias Abegg, Anke Salmen, Hilary Michelle Grabe

**Affiliations:** 1 Department of Ophthalmology, Inselspital, Bern University Hospital, University of Bern, 3010 Bern, Switzerland; 2 Department of Neurology, Inselspital, Bern University Hospital, University of Bern, Bern, Switzerland

**Keywords:** MOG, MS, Optic neuritis, RNFL, Ganglion cell layer, OCT

## Abstract

**Background:**

Myelin oligodendrocyte glycoprotein immunoglobulin G associated optic neuritis (MOG-ON) is a recently described entity. Recent studies have shown that MOG-ON has a more severe clinical presentation than classic optic neuritis (ON).

**Objective:**

This study aimed to define morphological characteristics of MOG-ON, correlate these with clinical characteristics and compare them with multiple sclerosis associated ON (MS-ON) and healthy controls (CTRL).

**Methods:**

In a retrospective study, we included MOG-ON and MS-ON patients seen between 2011 and 2018 at the University Hospital Bern. Data from clinical examination, perimetry, and optical coherence tomography (OCT) were analyzed.

**Results:**

A total of 66 eyes of 43 patients were included; 22 MS-ON and 33 CTRL eyes were sex- and age-matched to 11 MOG-ON eyes. We found significantly worse visual acuity at nadir, but better recovery and thinner global peripapillary retinal nerve fiber layer thickness in MOG-ON patients compared to MS-ON patients. Both groups exhibited irregular thinning of the macular ganglion cell layer. Furthermore, the visual acuity and visual field parameters correlated to retinal layer thickness only in MOG-ON eyes.

**Conclusion:**

In comparison to MS-ON, MOG-ON is associated with more prominent acute vision loss and more pronounced global thinning of the pRNFL. Both entities result in similar final visual acuity and atrophy of the macular ganglion cell layer.

## Introduction

The detection of myelin oligodendrocyte glycoprotein (MOG) antibodies in cases of optic neuritis (ON) has recently led to the differentiation of MOG immunoglobulin G associated optic neuritis (MOG-ON) from the more common idiopathic or multiple sclerosis (MS) associated optic neuritis (MS-ON) [1, 2]. Clinically, MOG-ON is characterized by a higher likelihood of bilateral disease, recurrent episodes, and involvement of the anterior optic nerve with associated disc edema compared to MS-ON [2–4]. Vision loss is often severe but with good recovery of vision [2]; recurrent episodes of MOG-ON can lead to poor visual outcomes in approximately a quarter of patients [2, 3].

Optical coherence tomography (OCT) is widely deployed in ophthalmology to quantify and monitor optic neuropathies, most frequently via the peripapillary nerve fiber layer (pRNFL). It has recently been shown that adding the macular ganglion cell layer (mGCL) thickness may have a better sensitivity than pRNFL alone in detecting optic neuropathies, and that the distribution of mGCL loss may provide additional localizing value of optic neuropathies [5, 6].

OCT characteristics of MOG-ON have to date been less well studied with sometimes conflicting results. Havla et al. found that ON eyes of MOG-antibody positive patients show reduced pRNFL thickness of the temporo-inferior und temporo-superior quadrants and reduced macular RNFL thickness in comparison to MS-ON [7]. In contrast, in comparison to MS-ON, Martinez et al. found no significant differences in the thickness of different retinal layers in six eyes with MOG-ON [8]. More recently, Sotirchos et al. found a decrease in thickness of the macular ganglion cell and inner plexiform layer (IPL) of MOG-ON as compared to MS-ON [9]. A small case series of three patients with MOG-ON reported preferential thinning of pRNFL of some quadrants [10], which is in contrast to the thinning of the temporal pRNFL quadrant typically seen in MS optic neuritis [11]. Altogether, these studies have to be interpreted with caution due to limited sample sizes, potentially explaining the conflicting findings.

This study aims to characterize the morphological features of MOG-ON in comparison to MS-ON and correlate these with visual function. Given the reported anterior involvement of the optic nerve in MOG-ON, the profound acute vision loss as compared to MS-ON and personal anecdotal evidence of a case that was initially misdiagnosed, we wondered whether MOG-ON shares morphological and functional features with anterior ischemic optic neuropathy. The latter is typically associated with an irreversible nerve fiber bundle loss. We hypothesized that MOG-ON may result in more retinal nerve fiber bundle defects than MS-ON resulting in atrophy predominantly localized in the distribution of the nerve fiber bundles. To investigate this hypothesis, we sought to determine if changes in thickness of the retinal layers were uniform across the macula or if the thickness varied particularly with respect to the horizontal raphe and the superior and inferior arcuate fibers of the macula.

## Methods

### Study design and patients

For this single-center retrospective study, we screened all patients examined at the Department of Ophthalmology, Inselspital, Bern, Switzerland between January 2006 and June 2018 for a diagnosis of MOG-ON or MS-ON by using the German translation of the keywords “neuritis” and “MOG” or “multiple sclerosis”. For the MOG group, eight patients were found with a total of 13 ON eyes; for the MS group, 95 patients with a total of 134 ON eyes. Inclusion criteria were confirmed diagnosis of MOG antibody associated disease (by antibody testing and neurologist) or MS (by neurologist) associated ON (diagnosed by an ophthalmologist) and availability of high resolution OCT images. There was no minimum follow-up for inclusion. Eyes with other relevant ocular diagnoses were excluded. In addition, a healthy control (CTRL) group was established by including available examination results of known healthy persons. Evaluation parameters for this study were visual acuity, visual field and retinal layer thicknesses (peripapillary and macular). This study was approved by the local cantonal ethics committee Bern under the project ID 2018-01151.

### MOG-immunoglobulin G testing

MOG status was tested using a cell-based assay (from 2015 until 04/2017: FACS assay, University Hospital Basel, Prof. Derfuss, Basel, Switzerland [12, 13] abbreviated as FACS; since 05/2017: commercial IIFT, Euroimmun AG, Lübeck, Germany, measured in-house, abbreviated as EUROIMMUN). The FACS assay results were classified as positive, borderline or negative. The in-house assay EUROIMMUN results were expressed as titers (< 1:10 negative, 1:10 borderline, > 1:10 positive).

### Clinical ophthalmic examinations

Affected eye laterality, number of ON attacks, monocular best corrected visual acuity (BCVA, transformed to LogMAR) and color vision using Ishihara-plates were collected for each patient for each visit. Mean visual field defect (MD) and the square root of Loss Variance (sLV) were determined from an Octopus 900 system with the EyeSuite software (Haag-Streit Diagnostics, Koeniz, Switzerland). Visual acuity corresponding to counting fingers and hand movements were rated as 1.854 and 2.301 in LogMAR values according to previously published quantitative data [14].

### Imaging/OCT

The OCT images (30° × 30°) of macular volume scans (20° × 20°) consisting of 49 line B-scans were taken with a Spectralis HRA OCT (Heidelberg Engineering, Heidelberg, Germany). They were exported from the OCT system software HEYEX which comes with the Spectralis HRA OCT using the included XML export tool. The pRNFL-thickness of each sector and global pRNFL was exported directly from the HEYEX which measured pRNFL thickness along a ring of 3.6 mm diameter centered on the disc. Segmentation of the macular volume scans was performed using OCTSeg (ARTORG Center, Inselspital, Bern, Switzerland) which is a custom established OCT Segmentation tool previously described [15–17]. The automated segmentation separated the retinal layers by determining the border between the internal limiting membrane (ILM), the ganglion cell layer (GCL), the inner nuclear layer (INL), outer nuclear layer (ONL), junction of the inner and the outer nuclear segments, the outer segment of photoreceptors/pigment epithelium complex and the Bruch membrane. Processed segmentation was checked by an experienced reader (RV), blinded to the group and manually corrected if required. Based on these borders, we determined the thickness of the combined GCL and IPL (GCIPL), the combined macular INL and outer plexiform layer (INOPL) and the complete retina thickness (CRT). An Early Treatment Diabetic Retinopathy Study (ETDRS) grid was centered over the fovea and the average thickness measurement values for each sector and cell layer were computed by the software and exported. Grayscale images of the thickness of retinal layers were generated by OCTSeg. The greyscale images corresponding to the GCIPL of all included study subjects were stacked with Fiji software [18], images of left eyes were flipped horizontally, and all images were manually aligned. Using the included tool ROIManager, a circular grid with five circles and 16 sectors for each circle was inserted over the stack, resulting in 80 regions of interest (Fig. 2) for which measurements of all images were performed and exported. All thickness values are shown in micrometers.

### Statistical analysis

Visual acuity as LogMAR, visual field values as MD and sLV and retinal layer thickness were evaluated. Mean values were compared using independent two-sample, one-sided Student’s *t* test for morphological parameters and an independent two-sample, two sided Student’s *t* test for the other parameters (Excel 2016, Microsoft Corporation). Correlation of parameters were computed with the Pearson test (MATLAB R2019, The MathWorks Inc.). Values are reported as mean ± standard deviation (SD) of the mean. To determine thickness steps, the GCIPL thickness values of horizontally mirrored sectors are divided by each other, in detail the higher value by the lower value of the two, to calculate the ratio. Three pooled sectors, representing nerve fiber bundles, were defined for a priori comparison with their mirrored counterpart; 17&18, 33&34 and 18&34. Figures were created using the export tool from HEYEX and Adobe Illustrator CC 2017 (Adobe Inc., San Jose, USA).

## Results

### Demographic and clinical characteristics of patients and CTRL

We found 11 eyes of six MOG antibody seropositive patients with confirmed optic neuritis and OCT images of good quality which could be used for the MOG group. The MS group consisted of 22 age and sex matched eyes of 17 MS patients, the CTRL group of 33 age and sex matched eyes of 20 healthy persons. Included patients were seen between 2011 and 2018. The two patient groups were comparable in age at time of onset of the ON, number of ON episodes and time between onset of ON and the last examination available. Visual field values were only available for nine eyes of the MOG group and 16 eyes of the MS group. Analysis including visual field values were only performed for eyes where the values were available.

Demographic and clinical characteristics of MOG-ON eyes, MS-ON eyes and CTRL are shown in Table 1.

**Table 1:** Demographics of MOG patients, MS patients and healthy controls (CTRL) with the corresponding measures of visual function

Group, number of eyes/variables	MOG *n* = 11	MS *n* = 22	CTRL *n* = 33	*p* value MOG/MS	*p* value MOG/CTRL	*p* value MS/CTRL
Age in years, mean (SD), [min:max]	26.25 (11.80) [10.28:46.56]	29.59 (9.13) [17.27:53.07]	26.30 (10.98) [9.13:46.59]	0.392	0.990	0.259
Female, *n* (%)	5 (45.5)	10 (45.5)	15 (45.5)	1.000	1.000	1.000
Age at first onset of ON in years, mean (SD), [min:max]	22.99 (11.79) [9.94:45.82]	26.54 (7.63) [15.82:46.06]	–	0.321	–	–
Number of ON episodes, mean (SD), [min:max]	1.18 (1) [1:2]	1.05 (1) [1:2]	–	0.211	–	–
Time between onset of ON and last examination in months, mean (SD), [min:max]	40 (33) [4:105]	40 (37) [1:130]	–	1.000	–	–
Last BCVA LogMAR, mean (SD)	0.24 (0.40)	0.28 (0.61)	− 0.07 (0.08)	0.831	**< 0.0002**	**0.0023**
BCVA at nadir, LogMAR (SD)	1.09 (0.66)	0.51 (0.75)	–	**0.043**	–	–
BCVA recovery at last examination, LogMAR (SD)	0.85 (0.74)	0.22 (0.46)	–	**0.007**	–	–
Last visual field, MD (SD)^a^	7.63 (5.17)	4.94 (4.50)	–	0.205	–	–
Last visual field, sLV (SD)^a^	4.53 (2.38)	4.23 (2.55)	–	0.782	–	–
Visual field recovery, MD (SD)^a^	1.91 (1.60)	1.92 (3.37)	–	0.995	–	–
Visual field recovery, sLV (SD)^a^	0.77 (0.93)	0.88 (1.56)	–	0.863	–	–

*BCVA* best corrected visual acuity, *MD* mean defect, *sLV* square root of loss variance, *SD* standard deviation

^a^MOG *n* = 9/MS *n* = 16

Bold values indicate statistical significance with a *p*-value less than 0.05

### Function: MOG-ON eyes vs. matched MS-ON eyes vs. CTRL eyes

In comparison to the MS group, the BCVA at nadir of our MOG group after ON was significantly worse, whereas the recovery of the visual acuity, defined as the difference between BCVA at nadir and BCVA at the last examination, was significantly better. This resulted in comparable mean BCVA on the final examination. The time between the examination at nadir and the last examination did not differ significantly between groups (Table 1).

Compared to the CTRL group, both patient groups had a significantly worse BCVA on the last examination. Visual field MD on the last examination was slightly higher in the MOG group than the MS group, but the difference was not significant (Table 1).

### Morphology: MOG-ON eyes vs. matched MS-ON eyes vs. CTRL eyes

**Fig. 1:**
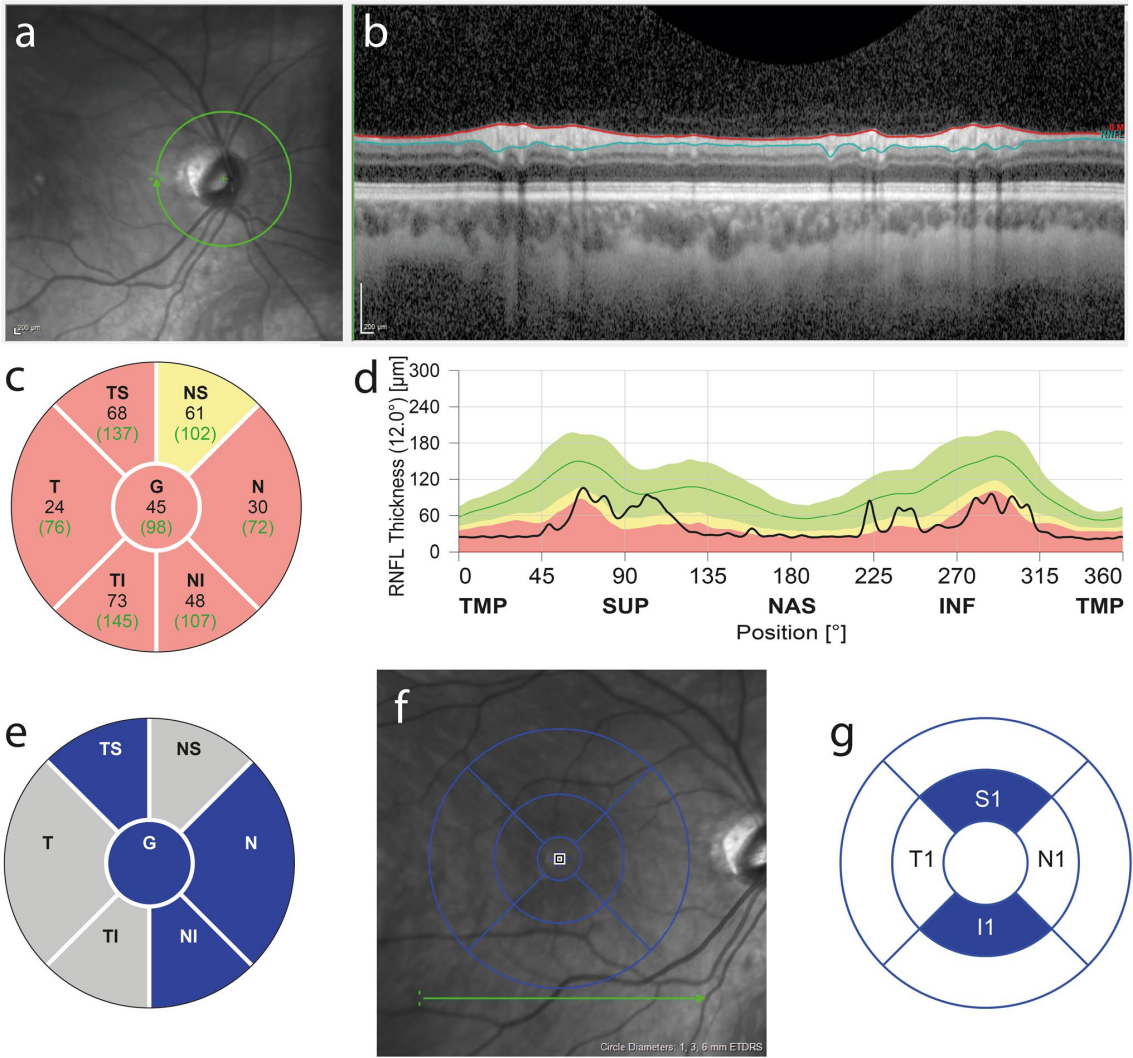
Clinical OCT output of a MOG-ON patient and differences in nerve fiber layer thickness. **a** Shows the OCT infrared scan of the optic disc and peripapillary region, **b** shows a vertical profile of the retina and the pRNFL corresponding to the location of the (green) ring in the OCT infrared scan. The two drawn lines represent the borders of the pRNFL. **c** Shows a sample of the mean thicknesses of the pRNFL of the different sectors whereas **d** shows the thickness profile of the pRNFL corresponding to the (green) ring and underlaid the normative values (green/top = normal, yellow/middle = borderline, red/bottom = pathologic). **e** Highlights the segments with significant differences in pRNFL thickness between MOG group and MS group, **f** shows an OCT infrared scan of the macula of a MOG-ON patient with an ETDRS grid overlay. **g** Highlights the significant differences in INOPL thickness in superior and inferior segments of the inner ring of the ETDRS grid when MOG and CTRL group is compared

In our study, the MOG group shows a significantly lower global pRNFL thickness compared to the MS group and the CTRL group. A significantly lower global pRNFL can also be seen for the MS group compared to the CTRL group. When pRNFL is split up into sectors, the MOG group shows significantly thinner pRNFL in the nasal, naso-inferior and temporo-superior sectors compared to the MS group (Table 2, Fig. 1).

**Table 2:** Morphology measurements for peripapillary retinal nerve fiber layer (pRNFL), ganglion cell and inner plexiform layer (GCIPL) and inner nuclear and outer plexiform layer (INOPL) of different regions of MOG patients, MS patients and healthy controls (CTRL)

Group, number of eyes/variables	MOG *n* = 11	MS *n* = 22	CTRL *n* = 33	*p* value MOG/MS	*p* value MOG/CTRL	*p* value MS/CTRL
pRNFL global, mean (SD)	59.45 (19.58)	75.27 (21.54)	102.85 (9.08)	**0.028**	**< 0.0001**	**< 0.0001**
pRNFL NS, mean (SD)	76.36 (28.41)	89.68 (31.15)	113.73 (18.04)	0.128	**< 0.0001**	**0.0004**
pRNFL N, mean (SD)	43.64 (17.71)	60.27 (21.84)	78.39 (10.95)	**0.021**	**< 0.0001**	**0.0001**
pRNFL NI, mean (SD)	65.55 (20.93)	85.59 (23.81)	117.88 (23.27)	**0.014**	**< 0.0001**	**< 0.0001**
pRNFL TI, mean (SD)	84.64 (30.28)	106.00 (40.32)	147.21 (20.98)	0.071	**< 0.0001**	**< 0.0001**
pRNFL T, mean (SD)	37.45 (13.59)	45.68 (15.40)	69.64 (8.46)	0.078	**< 0.0001**	**< 0.0001**
pRNFL TS, mean (SD)	86.91 (31.25)	110.59 (25.65)	147.82 (14.95)	**0.016**	**< 0.0001**	**< 0.0001**
GCIPL, mean (SD)	74.64 (10.95)	75.95 (10.27)	99.14 (4.83)	0.373	**< 0.0001**	**< 0.0001**
INOPL, mean (SD)	70.25 (3.10)	69.25 (0.92)	69.42 (0.75)	0.092	0.085	0.474
INOPL N1, mean (SD)	71.28 (9.28)	68.12 (3.75)	68.23 (3.16)	0.094	0.060	0.452
INOPL S1, mean (SD)	71.96 (3.30)	70.55 (0.37)	70.70 (0.27)	**0.032**	**0.020**	**0.047**
INOPL T1, mean (SD)	67.85 (1.91)	67.89 (1.86)	68.24 (1.84)	0.479	0.278	0.249
INOPL I1, mean (SD)	69.91 (0.81)	70.44 (1.15)	70.48 (0.29)	0.099	**0.001**	0.417

*N* nasal, *T* temporal, *I* inferior, *S* superior

Bold values indicate statistical significance with a *p*-value less than 0.05

**Fig. 2:**
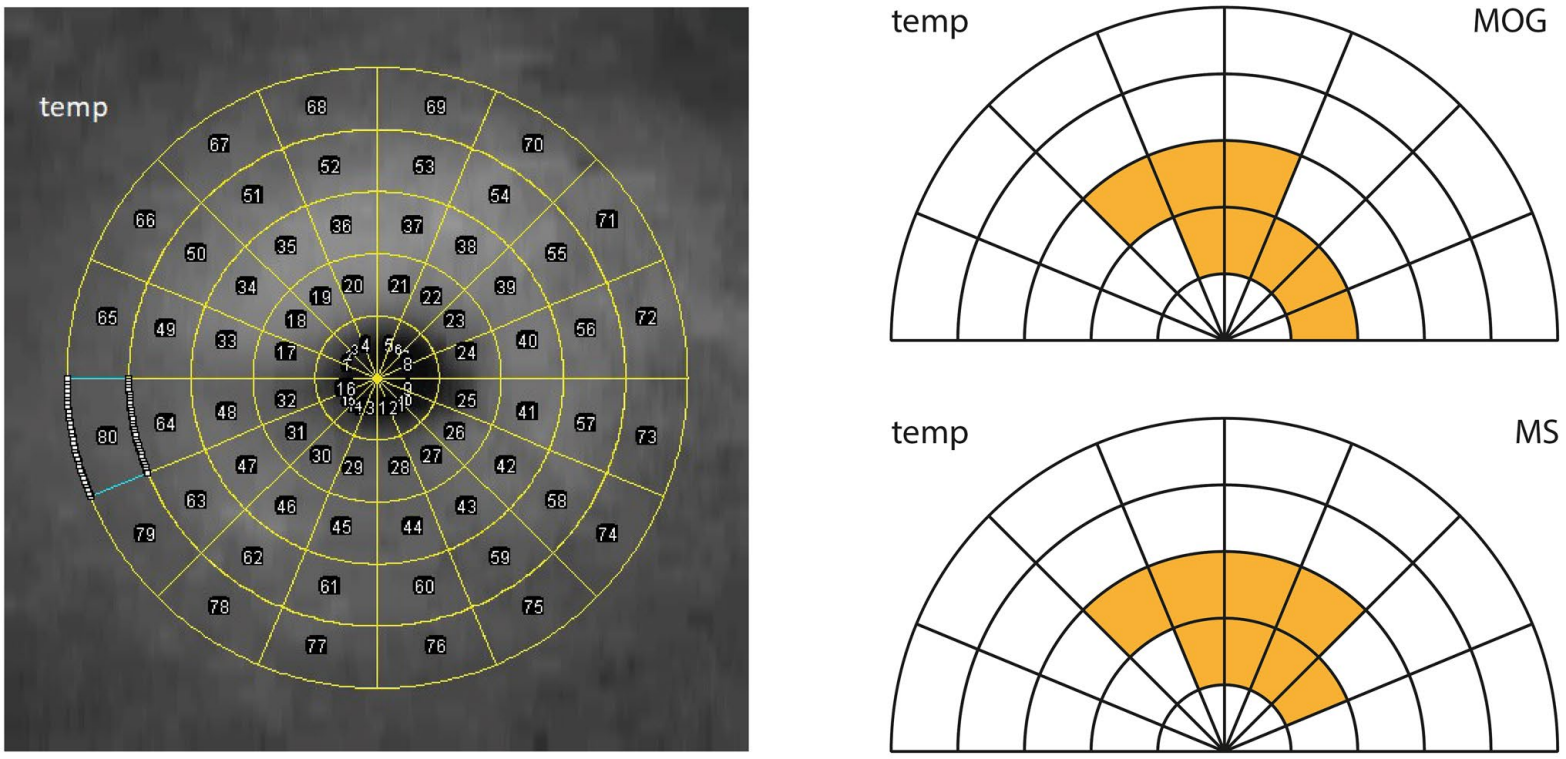
Regions of interest over the Macula for GCIPL comparison and thickness steps over the horizontal raphe. The left image shows an overlay of the OCT infrared scan of the macula and the grid with regions of interest used for this study. The graphics in the right part show the same regions of interest, highlighted are the regions which show significantly different ratios between its horizontally mirrored counterparts when MOG group and MS group respectively are compared to CTRL group. For simplification only the upper half is shown

GCIPL thickness of the inner ring of the ETDRS grid is significantly lower in both patient groups compared to the CTRL group without a significant difference between the two patient groups. A slightly higher INOPL thickness can be found in the MOG group compared to the CTRL group in the superior and a slightly lower INOPL thickness in the inferior inner sector (Table 2, Fig. 1). The a priori comparisons of the three pooled sectors and their horizontally mirrored counterparts do not show any significant thickness step differences over the horizontal raphe when the two patient groups are compared to each other. However, we found some significant differences between each patient group and CTRL group in other pooled sectors ratios to its mirrored counterparts (Fig. 2).

### Morphology-function: MOG-ab associated ON eyes vs. matched MS associated ON eyes

Eyes of the MOG group show an inverse correlation of the ratio of the ETDRS inner ring GCIPL thickness to CRT with visual function parameters such that thinning correlates with decreased function; the strongest and most significant can be found for the correlation of GCIPLr to visual field MD. When single sectors of the ETDRS grid inner ring (Fig. 1f, g) are compared to function parameters, the inferior sector shows the strongest negative correlation with *R*^2^ values > 0.4 for all three parameters (BCVA, visual field MD and sLV). In the MS group there is no significant correlation between GCIPLr and functional parameters (Table 3).

**Table 3:** Correlation analysis of OCT measures and visual function parameters: table depicts correlation coefficient/*R*^2^/*p* value and 95% confidence interval; ganglion cell and inner plexiform layer ratio to complete retina thickness (GCIPLr) of the inner ring of the ETDRS grid

Var1/Var2 corr	MOG	MS
GCIPLr BCVA	− 0.55/0.30/0.08 [− 0.86; 0.08]	− 0.23/0.05/0.30 [− 0.59; 0.21]
GCIPLr MD	**− 0.76/0.57/0.02 **[− 0.95; − 0.20]	− 0.12/0.01/0.66 [− 0.58; 0.40]
GCIPLr sLV	− 0.66/0.43/0.05 [− 0.92; 0.01]	− 0.23/0.05/0.40 [− 0.65; 0.30]

Bold values indicate statistical significance with a *p*-value less than 0.05

## Discussion

In this retrospective case series, we found a more severe vision loss at nadir in MOG-ON compared to MS-ON. The visual recovery, however, was significantly better in the MOG patients such that the final visual outcome was comparable between the two disease groups. Regarding morphological changes, we found a significant reduction of the peripapillary retinal nerve fiber layer and macular ganglion cell layer on the last examination in both disease groups as compared to healthy controls. The morphology of MOG and MS associated optic neuropathy seems relatively similar with some differences: MOG-ON shows a significantly thinner pRNFL in the overall pRNFL thickness which is likely driven by significantly thinner pRNFL in the superior and inferior parts of the pRNFL, while the temporal thinning seems similar in MS-ON and MOG-ON. These findings seem to parallel the functional findings with worse visual fields in MOG (although not statistically significant) and equal or even slightly better visual acuity (also not statistically significant) in MOG-ON as compared to MS on the last examination. The GCIPL thickness in contrast seems equal in both disease groups, which corresponds to the equal visual acuity on the last examination. Taken together this data suggests a more profound optic neuropathy after a single episode of MOG-ON compared to MS-ON and a tendency to affect the visual field more than the visual acuity as a long-term outcome. As a potential OCT correlate, MOG-ON seems to affect the superior and inferior portions of the optic nerve more than MS-ON, while involvement of central (i.e., macular) ganglion cells and visual acuity is similar in the two disease groups.

We could not confirm our initial hypothesis of an asymmetric nerve fiber bundle loss in one or the other disease group, i.e., we found that the vertical asymmetry, which is a hallmark of ischemic optic neuropathy with its associated nerve fiber bundle loss, is not present or not different in MS-ON and MOG-ON. Although our data indicate a more profound involvement of superior and inferior nerve fiber bundles in MOG-ON, there was no significant vertical asymmetry in this involvement. This argues against a vascular component of the optic neuropathy as seen in anterior ischemic optic neuropathy where differences in superior and inferior GCIPL layer thickness have been described [19, 20].

Regarding OCT characteristics, our study confirms previous findings of a significantly decreased thickness in global pRNFL of MOG-ON eyes in comparison to healthy eyes as well as MS-ON eyes [7]. The more pronounced thinning of the pRNFL may be a consequence of more profound inflammation in the acute disease phase and correlate with the worse vision in the acute phase of MOG-ON. The GCIPL ETDRS inner ring thickness did not differ significantly between the MOG and MS group despite the significant difference in pRNFL thickness between the groups. This might be explained by the distribution of pRNFL thinning in MOG-ON: the temporal-superior, nasal and nasal-inferior segments are more affected in MOG-ON than MS-ON, but both entities show similar thinning in the temporal pRNFL segment, which is comprised of nerve fibers heading to the macula, resulting in similar macular GCIPL thickness. These results may correspond to published data by Havla et al. who showed more severe thinning in the temporal inferior and temporal superior sectors when comparing MOG-ON to MS-ON but no significant difference in the temporal sector [7]. However, Sotirchos et al. found differences in the temporal pRNFL comparing MOG-ON to MS-ON and in thinning of GCIPL between these two groups, highlighting the conflicting findings in the literature due to limited sample sizes in all studies [9]. As temporal pRNFL thickness of MS-ON eyes does not differ significantly from temporal pRNFL thickness in neuromyelitis optica spectrum disorders (NMOSD) [11], the latter of which are known to result in severe atrophy [21] the similar outcome between temporal pRNFL thickness in MOG-ON and MS-ON may be due to relatively more severe temporal thinning in MS-ON rather than less severe temporal thinning in MOG-ON. This similarity in temporal pRNFL thickness corresponds to the similar BCVA at last examination between the two patient groups.

The correlation of morphology and function was clearer in the MOG group. Each visual function parameter (BCVA at last examination, MD of the visual field and sLV of the visual field) showed a negative correlation for the ratio of the GCIPL to CRT, which supports the correlation between OCT findings and visual field loss as well as BCVA described in smaller cohorts [22]. This correlation may have future implications for prognosis in patients with MOG-ON whereas the morphological findings in MS-ON appear less predictive of visual function. These findings are consistent with the lack of correlation between GCL thickness and high contrast visual acuity despite correlation of GCL thickness with low contrast visual acuity previously described in MS-ON [23, 24].

We found a worse visual acuity at nadir in the MOG-ON group in our cohort with a worst BCVA around one. This result supports the findings of Jarius et al. who found over two thirds of MOG-ON patients presenting at least once with BCVA worse than one [2]. The underlying reason for the better recovery of vision is still unclear; a potential correlate to decreasing MOG-antibody serum concentrations with reduced inflammatory activity in remission following the acute phase of the disease is thus far speculative [4, 12, 25]. Our study did not show a significant difference in the number of bilateral cases of optic neuritis in the MOG group compared to the MS group; similarly, we did not show a significant difference in the number of recurrent episodes. These findings are likely due to a small sample size of 11 eyes in the MOG group and not a refutation of these previously reported characteristics [2]. The results are summarized in Table 4 and shown in Fig. 1 graphic “*e*” and “*g*” and Fig. 2.

**Table 4:** Summary of the findings

Differences in MOG-ON compared to MS-ON	Similarities between MOG-ON and MS-ON
Significantly thinner global pRNFL in MOG-ON	Similar thinning of the temporal pRNFL
Inverse correlation of macular ganglion cell layer thickness with visual function in MOG-ON	Similar thinning of the macular ganglion cell layer
Worse visual acuity at nadir in MOG-ON	Similar visual acuity at last examination

Our study has several limitations. Most importantly, our sample size is small and larger studies are needed for corroboration of our findings. An additional important limitation is testing bias in the diagnosis of MOG-ON; as this entity is only recently described, testing for MOG-IgG antibodies may have predominantly been performed only in severe or bilateral cases of optic neuritis. This bias in testing would skew our analysis towards more severe cases in the MOG-ON group, with correspondingly more severe OCT findings. In addition, although follow-up time is similar between groups, it is still relatively short in terms of a chronic disease such that recurrent disease is likely underrepresented in our study. Furthermore, vision before ON was not documented and some eyes may have an undiagnosed pre-existing ON. We were not able to include cases of NMOSD eyes in our study, which would be an additional cohort of interest.

## Conclusion

MOG-ON should be considered in all patients with ON with an atypical or more severe clinical course. MOG-ON was associated with more prominent acute vision loss than MS-ON but resulted in similar final visual acuity. In comparison to MS-ON, we found a significantly decreased thickness in global pRNFL in MOG-ON and no significant difference in the GCIPL thickness. The GCIPL thickness showed a significant inverse correlation with MD in visual fields in MOG-ON, but not MS-ON, which may have future prognostic implications in MOG-ON.

## Data Availability

Access to anonymized patient data will be granted on request.
